# Isolation and genomic characteristics of the novel variant infectious bursal disease virus in China

**DOI:** 10.3389/fvets.2023.1314903

**Published:** 2023-12-11

**Authors:** Zongyi Bo, Shixu Wang, Keke Xu, Chengcheng Zhang, Mengjiao Guo, Yongzhong Cao, Xiaorong Zhang, Yantao Wu

**Affiliations:** ^1^Joint International Research Laboratory of Agriculture and Agri-Product Safety, The Ministry of Education of China, Yangzhou University, Yangzhou, China; ^2^College of Veterinary Medicine, Jiangsu Co-innovation Center for the Prevention and Control of Important Animal Infectious Disease and Zoonoses, Yangzhou University, Yangzhou, Jiangsu, China

**Keywords:** IBDV, novel variant strain, genomic characteristics, phylogenetic analysis, antigenic drift

## Abstract

The infectious bursal disease virus (IBDV) is a member of the viruses that can induce immunosuppression in chickens. In recent years, more and more IBDV-infected cases by the novel variant IBDV were reported in China, and it has been demonstrated that currently used vaccines could not provide complete protection against these new IBDV variants. However, a lack of comprehensive analysis of the genomic characteristics of the novel variant strain IBDV has hampered its vaccine development. In this study, a strain of IBDV, designated HB202201, was phylogenetically analyzed, and it was found that the hypervariable region (HVR) of VP2 belonged to the novel variant strain. Furthermore, the 5′- and 3′-ends of segments A and B were analyzed using the rapid amplification of cDNA end (RACE) method. After the full-length of segment A and segment B were determined, the phylogenetic analysis of the segment A and segment B showed that the isolated HB202201 belonged to A2dB1 genotype, which demonstrated the HB202201 belonged to the novel variant strain. In addition, the specific mutations in VP1-VP5 amino acids were analyzed, which showed that there were multiple typical mutations in novel variant IBDV proteins, including VP1 (G24, I141, V163, and E240), VP2 (K221, and I252), VP3 (Q167 and L196), and VP5 (R7, P44, R92, G104, and E147), whereas there was no typical mutation in VP4. This study provides insights into the genomic and antigenic characteristics of the novel variant IBDV, which will promote the development of novel vaccine against the novel variant IBDV.

## 1 Introduction

Infectious bursal disease (IBD), also known as Gumboro disease (GD), continues to pose a major threat to the poultry industry, even though it has been observed since 1962, when it was first reported in the United States (1). Infectious bursal disease virus (IBDV), the causative agent of IBD, belongs to the *Birnaviridae* family and *Avibirnavirus* genus (2). IBDV is the smallest segmented RNA virus in animals with a bisegmented dsRNA genome, including segment A and segment B (3, 4). Totally five proteins can be encoded by the IBDV. The segment A, about 3.2 kb in length, can encode VP5, VP2, VP3, and VP4 (5, 6). The segment B, about 2.8 kb in length, just encodes VP1 protein (7). The B lymphocytes in the bursa of Fabricius (BF) are the main target of IBDV, which further leads to the immunosuppression in young chickens, especially the chickens at 3–6 weeks when the BF reaches maximum development. Despite causing direct tissue injury to the infected chickens, IBDV infection can also reduce the immune response of chickens to vaccination. Meanwhile, the IBDV infected chickens will be more susceptible to infection by other pathogens (2, 8).

There are two serotypes of IBDV, serotype I and serotype II, and there is no cross reaction between them (9). Serotype I IBDV is pathogenic to chickens, while the serotype II IBDV is usually isolated from turkeys and is avirulent in chickens (10, 11). There are still many genotypes of IBDVs in serotype I IBDV. Based on their virulence, the serotype I IBDVs can be divided into very virulent IBDV (vvIBDV), classical virulent IBDV (clIBDV), antigenic variant IBDV (vaIBDV), and attenuated IBDV (atIBDV) (12). Different from the American variant strain, the novel variant IBDV isolated in China showed with < 97.7% (VP1) or 98.7% (VP2) amino acid sequence identity, and has circulated in China since 2017 (13). Since then, more and more novel variant IBDV strains have been reported in China, resulting in huge economic losses to Chinese poultry industry (14, 15).

Vaccination is the method mostly commonly used for the control of IBDV. However, mutations in IBDV-encoded proteins and reassortment between segment A and segment B among different strains contribute to antigenic drift among different genotypes of IBDVs (8, 16). It has been reported that two substitution mutations (D318G and E323D) in the HVR of VP2 are an important factor in the escape of the variant DelE IBDV strain from neutralizing monoclonal antibodies (17). Previously, it has been reported that the natural homologous recombination between live vaccines and wild-type IBDV strains has also been reported, which highlights the risk of using live attenuated vaccination for IBDV control and reminds us to use live IBDV vaccines more cautiously (18–20). More seriously, it has also been demonstrated that the currently used vaccine cannot provide full-protection against the novel variant IBDV (21). Therefore, it is necessary to comprehensively study the genomic and antigenic characteristics of the novel variant strain, so that a new useful vaccine against the novel variant IBDVs can be developed.

In this study, a strain of IBDV, designated HB202201, was isolated from the young laying hens. Phylogenetic analysis of the HVR region of VP2 showed that it belonged to the novel variant IBDV. Then, the full-length sequences of segment A and segment B were sequenced and phylogenetically analyzed, which showed HB202201 belonged to A2dB1 genotype. The specific mutations in IBDV-encoded proteins and the antigenic index of the HVR of VP2 were analyzed, which showed that there were multiple specific mutations and antigenic regions in the novel variant IBDV. This study analyzed the genomic characteristics of the novel variant IBDV, and lays the foundation for its molecular study and vaccine development.

## 2 Materials and methods

### 2.1 Samples

Samples of bursa of Fabricius (BF) were collected from a poultry farm located in Hebei Province in China in 2022. Total 2–4 g of collected samples were ground in a mortar and put into freeze-thaw three times. The mixture was then centrifuged at 3,600 rpm for 10 min at 4°C, and the supernatants were used to extract the RNA.

### 2.2 RT-PCR

Total RNA of the collected clinical samples was extracted using TRIzol Reagent (CWBIO, Taizhou, China) and reverse transcribed into cDNA using EasyScript Reverse Transcriptase (TransGen Biotech, Beijing, China). The primers used in detecting the existence of IBDV were listed below, sense primer IBDV-HVR-F: 5′-TGACAGCAGTGACAGGC-3′; anti-sense primer IBDV-HVR-R: 5′-ACTCGTTCGTAGGCCAC-3′. The PCR products were analyzed by gel electrophoresis on a 1.5% agarose gel, and the PCR product with an expected band size of 450 bp was considered as IBDV VP2 positive. The purified DNA was then sent for direct Sanger sequencing (Sangon, Shanghai, China).

### 2.3 Virus propagation

Eleven-day-old SPF chicken embryos (Beijing Boehringer Ingelheim Vital Bio, Beijing, China) were used for viral isolation and propagation. The homogenates of the PCR-positive samples were passed through a 0.45 μm filter and 200 μL of the supernatant was inoculated into the SPF chicken embryos through chorioallantoic membrane (CAM) as previously described (22). The embryo was examined daily for survival by candling. The dead embryo within 24 h was discard. Finally, after 5 days incubation, the chorioallantoic membrane was harvested and RT-CPR was used for the detection of IBDV.

### 2.4 Determination of the ends of segment A and segment B

The sequences of the 5′ and 3′ ends were determined using rapid amplification of cDNA ends (RACE) method using a HiScript-TS 5′/3′ RACE Kit (Vazyme Biotech, Nanjing, China) (23, 24). The 5′ or 3′ end of PCR primers specific to the IBDV segment A and segment B genes were designed by aligning sequences for each gene from GenBank and targeting conserved regions within these sequences. The specific primers used were designed by our lab and listed in Table 1. After electrophoresis, the suspected positive bands were cloned to the pEASY-Blunt cloning vector (TransGen Biotech, China). Three positive clones for each product were sent to Sangon Biotech for sequencing.

**Table 1:** The primers for amplification of the sequences of IBDV.

**Name**	**Sequence**
IBDV-5GSP-A	5′-CGTTGATGTTGGCTGTTGCAG-3′
IBDV-5GSP-B	5′-GCGATGTCTGGCGGGTACGCA-3′
IBDV-3GSP-A	5′-GCCAACTTTGCACTCAGCGAC-3′
IBDV-3GSP-B	5′-ACTCGACGAGTTCCTAGCCGA-3′
SegA-F	5′-GAGCCTGAGTGAACTGACAGA-3′
SegA-R	5′-CGCTTGTGGTGCGTTTGCAAG-3′
SegB-F	5′-ACTACCCAACACACCGCCCTA-3′
SegB-R	5′-CGAATGCCTCACCGAACTCTG-3′

### 2.5 Determination of the full-length sequences of segment A and B

The primers for the full-length sequences of the segment A and segment B were designed based on the sequences assembled from the 5′-RACE and 3′-RACE sequencing results (Table 1). The amplified PCR products were excised and sequenced. The GenBank accession number of the full-length sequences of segment A and segment B of HB202201 were OR290134.1 and OR500109.1, respectively.

### 2.6 Sequence alignment and phylogenetic analysis

The amino acids of the VP1-VP5 of HB202201 were aligned using the MegAlign module in DNASTAR Lasergene 7 (DNASTAR Inc., Madison, WI, USA). The phylogenetic trees of the HVR of VP2, segment A, and segment B were constructed using MEGA-X with the maximum likelihood method (ML). The ML tree was constructed using MEGA-X software with the General Time-Reversible, Gamma Distribution Substitution Model and 1,000 Bootstraps. The strain name, country, year of collection, and the GenBank accession number of each IBDV strain were labeled in the phylogenetic trees.

### 2.7 Antigenic index analysis

The amino acid sequences of the HVR of the VP2 from the representative different genotype strains were downloaded and the antigenic index was calculated using the Jameson-Wolf algorithm in the software DNASTAR Lasergene 7 (25).

## 3 Results

### 3.1 Isolation and phylogenetic analysis of HB202201

**Figure 1:**
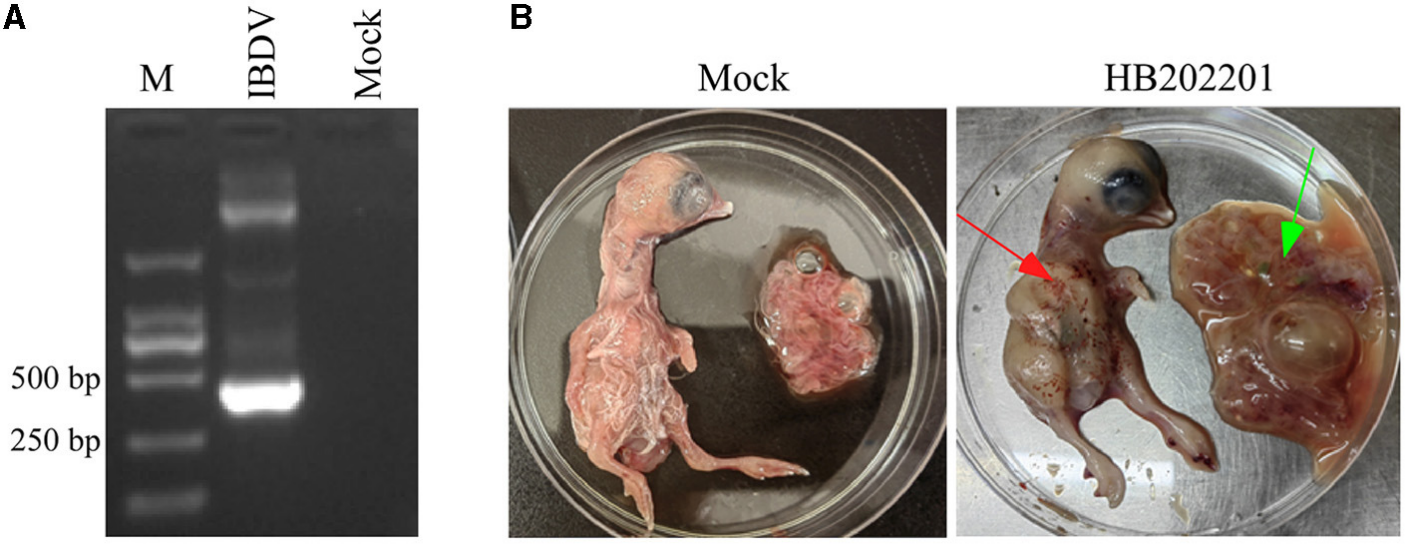
Detection and isolation of HB202201 IBDV. **(A)** The RT-PCR result of the detection of the existence of IBDV. **(B)** The eleven-day-old SPF chicken embryos were inoculated with isolated HB202201 through chorioallantoic membrane (CAM) or PBS as control group. After 5 days incubation, the clinical signs of the embryos were checked (The red arrow showed the hemorrhage in chicken embryos, and the green arrow showed the oedema in CAM).

**Figure 2:**
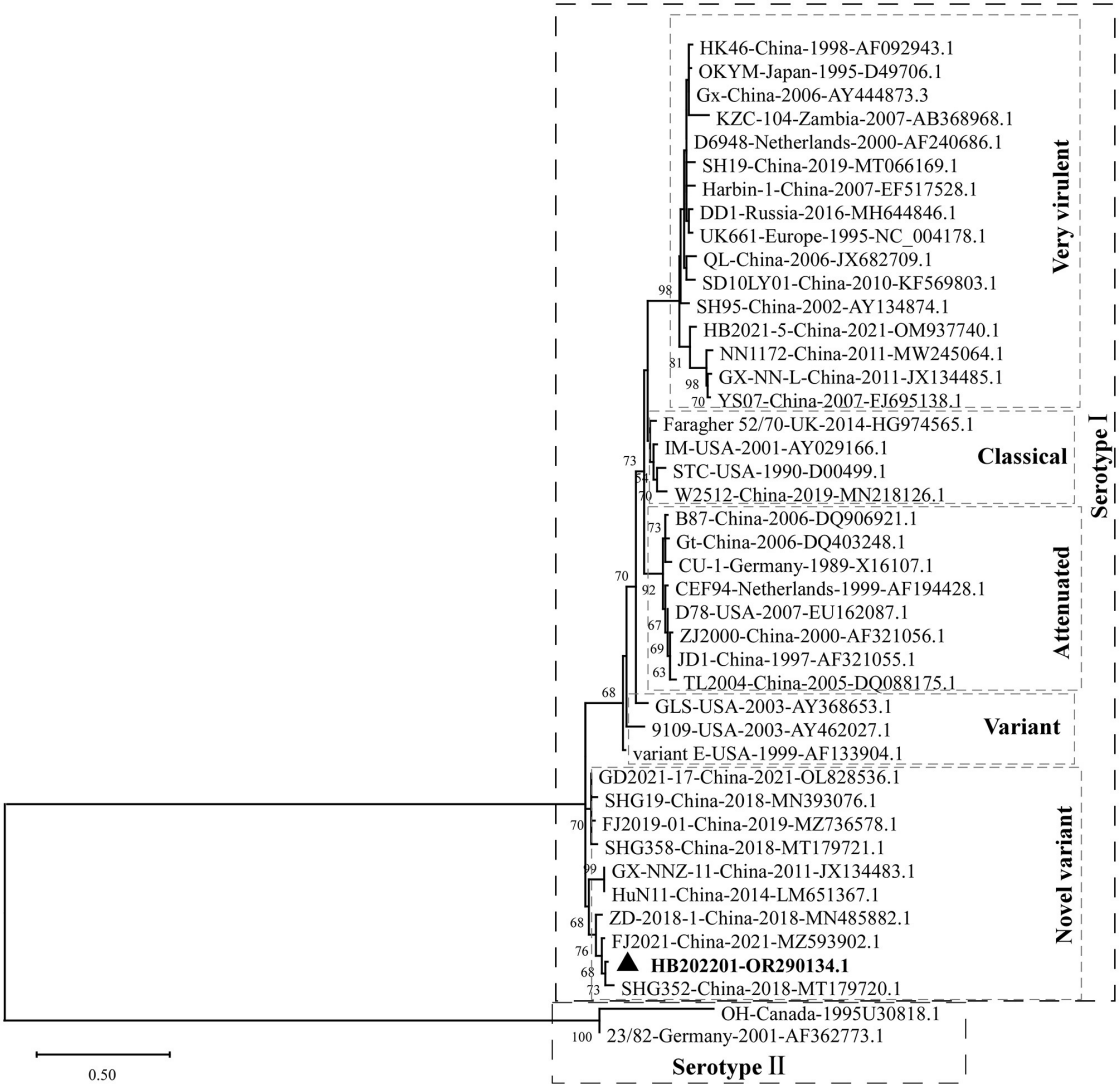
Phylogenetic analysis of the HVR of IBDV VP2. The HVR of VP2 in different IBDV strains, including very virulent strain, variant strain, novel variant strain, classical strain, attenuated strain of serotype I, and two serotype II stains, were analyzed to construct the maximum likelihood (ML) tree using MEGA-X software.

The clinical samples from the young laying hens with suspected IBD were collected and the existence of IBDV was checked using RT-PCR. The results showed that the IBDV was positive (Figure 1A), which was designated as HB202201. Then, the supernatant from the positive sample was inoculated into chicken embryos through CAM for IBDV isolation. After incubation for 5 days, the results showed that there was no death in IBDV incubated chicken embryos. However, the dwarfed carcass with hemorrhage was found in IBDV infected-chicken embryos, as well as the oedema and hemorrhage in CAM (Figure 1B). The HVR sequences (616 nt to 1050 nt) in VP2 gene, which contributes to the antigenic variation and virulence of IBDV, is commonly used for IBDV genotype classification (26, 27). In this study, to determine the genotype of the HB202201, the HVR of VP2 was firstly sequenced and phylogenetically analyzed. The phylogenetic result of the HVR of different IBDVs showed that HB202201 belonged to the novel variant IBDV (Figure 2). Meanwhile, the amino acid sequence alignment of the HVR of different novel variant IBDVs isolated in China was performed. The results showed that the HVR of novel variant IBDV was highly conserved, with only two amino acid mutations observed in two of the novel variant IBDVs (Supplementary Figure S1).

### 3.2 Sequencing of the full-length of the isolated novel variant IBDV

**Figure 3:**
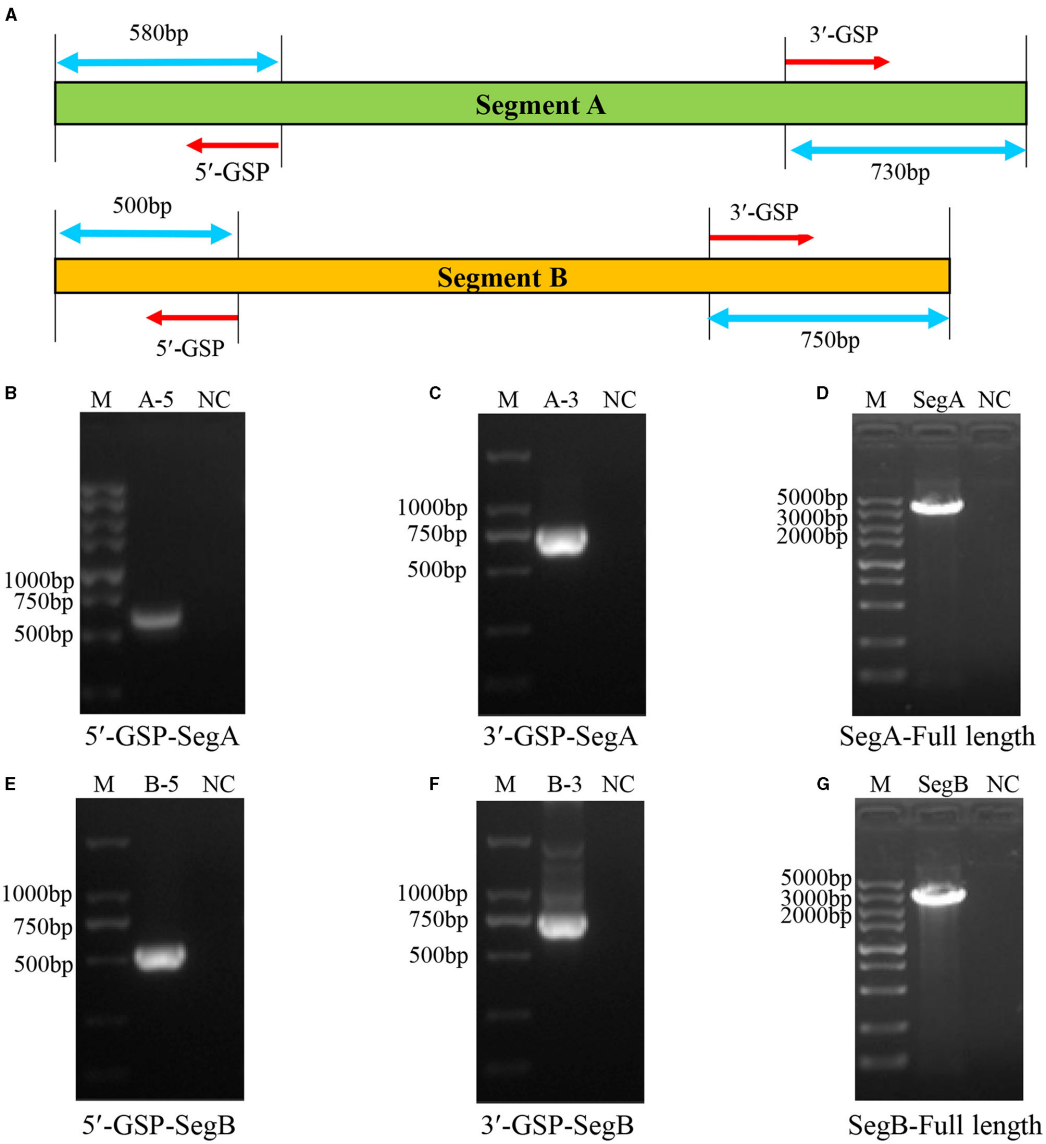
Amplification of the full-length of HB202201 segment A and segment B. **(A)** The schematic of the RACE method to determine the 5′ and 3′ sequences of the segment A and segment B. Briefly, four gene-specific primers (GSPs) which targeted the conserved sequences of IBDV segment A and B were designed, combining with universal primers, the 5′ ends and 3′ ends sequences of the segment A and segment B were amplified. **(B)** The PCR result of the 5′ end of segment A. **(C)** The PCR result of the 3′ end of segment A. **(D)** The PCR result of the full-length sequences of segment A. **(E)** The PCR result of the 5′ end of segment B. **(F)** The PCR result of the 3′ end of segment B. **(G)** The PCR result of the full-length sequences of segment B.

Before the amplification of its complete segment A and segment B, the 5′ and 3′ end sequences of them were determined using RACE method. The 5′- and 3′-ends of segment A and segment B were amplified as shown in Figure 3A. The results showed that the 5′ ends (Figure 3B) and 3′ ends (Figure 3C) of segment A, and the 5′ ends (Figure 3E) and 3′ ends (Figure 3F) of segment B were all successfully amplified. Full-length primers for segment A and segment B were designed according to the sequencing results, and the PCR results showed that both of full-length of segment A (Figure 3D) and segment B (Figure 3G) were successfully amplified. After sequencing, the results showed that the full-length of segment A was 3260 bp and segment B was 2827 bp, respectively.

### 3.3 Phylogenetic analysis of segment A and segment B of HB202201

**Figure 4:**
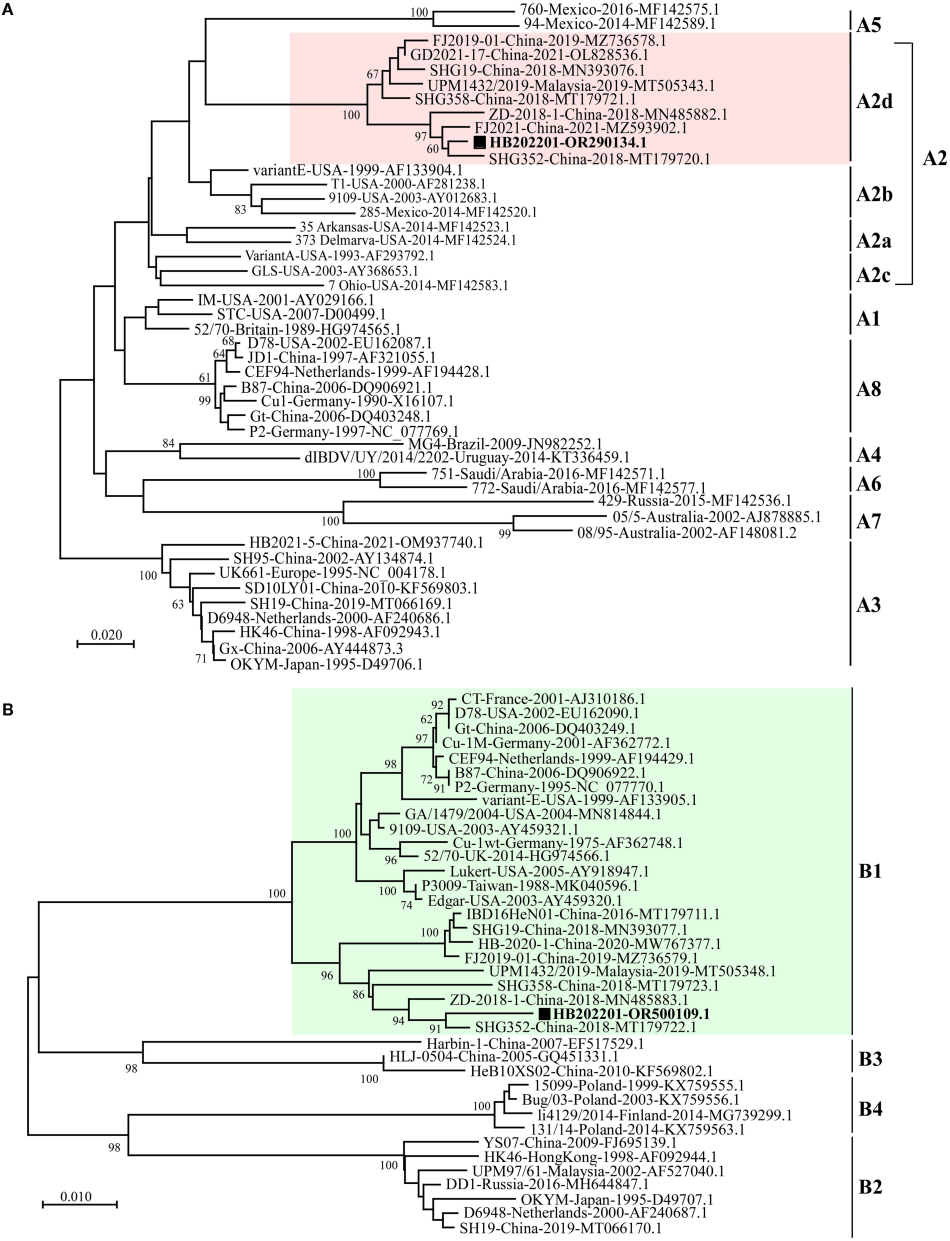
HB202201 belonged to the genotype of A2dB1. **(A)** The HVR sequences in different IBDVs were analyzed to construct the maximum likelihood (ML) tree using MEGA-X software. **(B)** B-Marker sequences of IBDV VP1 in different IBDVs were analyzed to construct the maximum likelihood (ML) tree using MEGA-X software.

To understand the phylogenetic characteristics of the newly isolated HB202201, the genotype of it was analyzed using the new classification method, in which the segment A of serotype I IBDV was classified into eight genotypes [A1, A2 (further classified into four lineages, A2a, A2b, A2c, and A2d), A3, A4, A5, A6, A7, and A8] and segment B was classified into B1, B2, B3, and B4 (28–30). The phylogenetic analysis of the HVR of VP2 showed that segment A of the newly isolated HB202201 belonged to A2d genotype (Figure 4A). Meanwhile, the phylogenetic analysis of segment B was also analyzed by using its phylogenetic marker (B-Marker) sequences of VP1, the results showed that the segment B of HB202201 belonged to B1 cluster (Figure 4B). Collectively, these data demonstrated that the isolated IBDV HB202201 strain belonged to genotype A2dB1. Additionally, the phylogenetic analysis of HB202201 was also performed using the full-length of segment A and segment B, both the phylogenetic results of segment A and segment B showed the HB202201 belonged to the novel variant IBDV (Supplementary Figure S2). Taken together, all these data demonstrated that the IBDV isolated in this study belonged to novel variant IBDV.

### 3.4 Amino acid characteristics of HB202201

**Figure 5:**
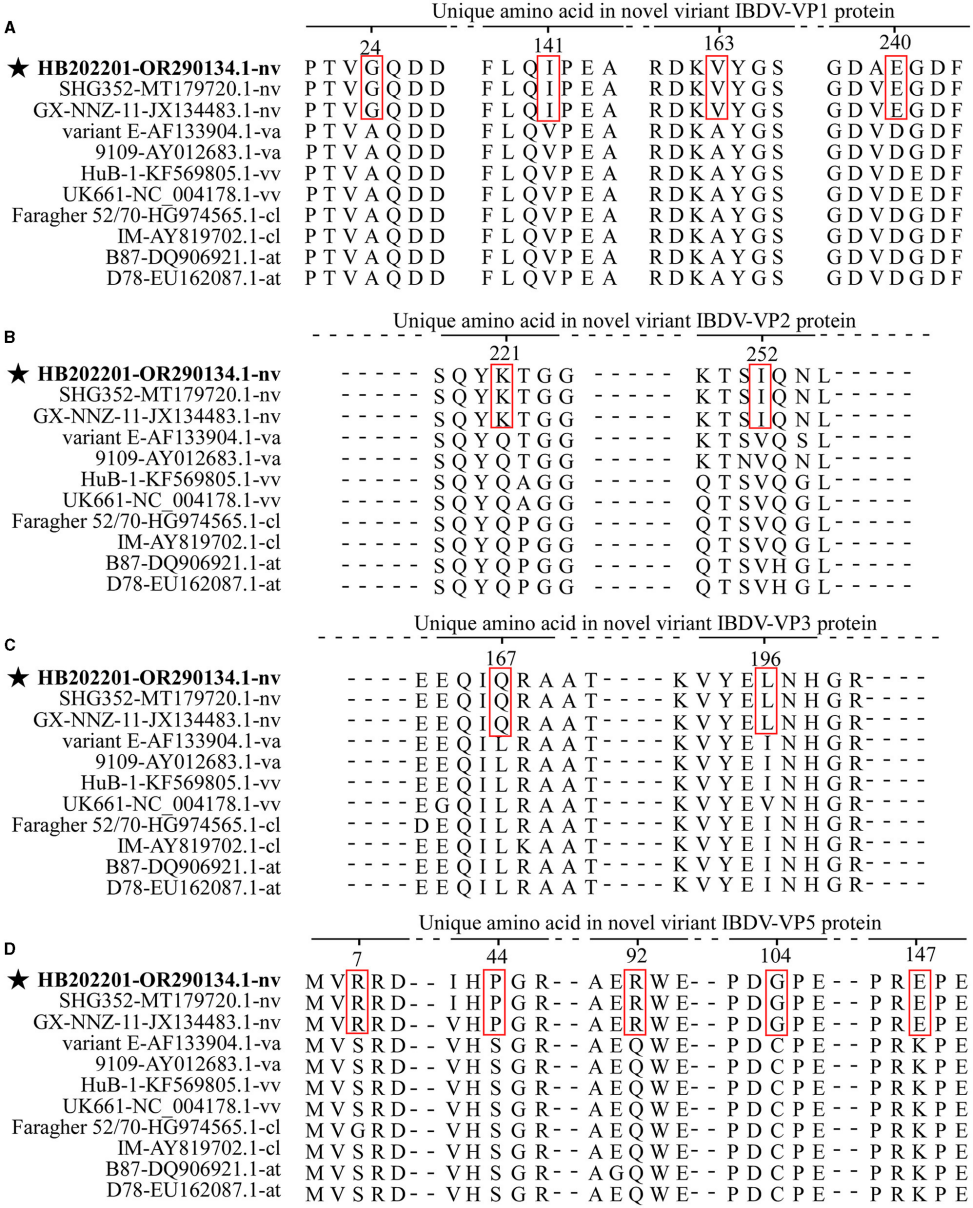
The amino acid mutations of the novel variant IBDV compared with other genotypes. Eleven representative strains of IBDV with different genotypes were chosen for the analysis of the typical mutations in the amino acid of VP1 **(A)**, VP2 **(B)**, VP3 **(C)**, and VP5 **(D)**. The sequences were aligned by the Clustal W method in DNASTAR software.

Mutation of the amino acid is an important factor that contributes to IBDV antigenic drift, pathogenic phenotyping, and cell tropism (31, 32). Previously, the amino acid comparison was used to define the characteristic of the isolated IBDV and it was found that there were specific amino acids in the novel variant IBDVs (33). In this study, to explore whether there were typical amino acid mutations in novel variant IBDVs, 11 representative strains of IBDV with different genotypes were analyzed. The alignment of the VP1, the viral RNA-directed RNA-polymerase protein encoded by segment B, showed four typical mutations (G24, I141, V163, and E240) (Figure 5A). The proteins encoded by segment A were then analyzed, and the results showed that there were two typical mutations (K221 and I252) in VP2 (Figure 5B), two typical mutations (Q167 and L196) in VP3 (Figure 5C), and five typical mutations (R7, P44, R92, G104, and E147) in VP5 (Figure 5D), while there were no typical mutations in VP4 protein. All these data demonstrated that there were multiple typical amino acid mutations in the novel variant IBDV.

It has been reported that the VP2 protein is the major antigenemic protein that can induce the neutralizing antibody against IBDV (16, 34). In this study, to determine whether there was antigenic drift in the novel variant strain, the antigenic index of the HVR of VP2 were analyzed using the Jameson-Wolf algorithm method in DNASTAR software. As shown in Supplementary Figure S3, compared with variant strains, such as variant E and 9019, the novel variant IBDV had just one region (5–20 aa) with a different antigenic index. Additionally, compared with vvIBDV, clIBDV, and atIBDV, the novel variant IBDV had three different antigenic index regions, including region 1 (5–20 aa), region 2 (36–45 aa), and region 3 (70–87 aa). These findings might help to explain why the currently used vaccines do not provide optimal protection against the novel variant IBDV.

## 4 Discussion

IBDV is one of the most important immunosuppressive pathogens affecting the poultry industry. The novel variant IBDV was first reported in China in 2017, since then, it has been circulated in most Chinese regions, and the happening rate of novel variant IBDV has increased year by year (13, 19, 35). Meanwhile, the infection of IBDV could significantly suppress the immune response after vaccination. It was reported that the novel variant strain could significantly suppress the antibody titer in both broilers and layers immunized with Newcastle disease virus (NDV) vaccine (36). Moreover, immunosuppression in IBDV-infected chicken makes it more susceptible to other pathogens, including bacteria and viruses (37). Therefore, there is a need to know the genomic and antigenic characteristics of the novel variant IBDV.

Variations in mortality rates have been revealed to be dependent on the strains of IBDV. Several studies have shown that the morality of IBDV varies from 1 to 50% in young chickens during the classical form of the outbreaks (2). However, for the novel variant IBDV in China, the infected chickens of 16-day-old showed no mortality and no gross symptoms (13). In this study, the BF sample was collected from the chickens with no obvious clinical symptoms, however, the dissection of them showed the dropsy of the BF. Then, IBDV was detected using RT-PCR and the results showed that the IBDV was positive. Chicken embryo incubation showed that it could lead to severe hemorrhage in the body of chickens, while there was no death of incubated chicken embryos (Figure 1).

To know the genotype of the newly isolated HB202201, the HVR of VP2, in which the mutations could lead to the alternation in virulence, tissue tropism, and antigenicity, was first phylogenetically analyzed (32, 38). The phylogenetic results of the HVR showed that the isolated HB202201 strain belonged to the novel variant IBDV (Figure 2). Next, to know the genomic characteristics of the full-length of segment A and segment B of HB202201, the complete sequences of it were sequenced. Till now, multiple methods can be used to determine the full-length of viruses, including Sanger sequencing and next generation sequencing (39, 40). In this study, the RACE method combining with Sanger sequencing was firstly used to determination of the 5′- and 3′- end of both segment A and B. This method was more accurate than the next generation sequencing, which might result in some gaps in the sequencing results. After the full-length sequences of segment A and segment B were successfully determined, the genotype of the HB202201 was analyzed using the new classification method in which the segment A was classified into eight genotypes (A1–A8) and segment B was classified into B1–B4. The results showed that HB202201 was A2dB1genotype (Figure 4), which belonged to a cluster of the novel variant group.

Previous studies have demonstrated the relationships between amino acid mutations with the virulence, replication capacity, cell tropism, and pathogenesis of IBDV (32, 41, 42). In this study, typical mutations in the VP1-VP5 proteins of the novel variant IBDV were analyzed by comparing with other vvIBDVs, vaIBDVs, clIBDVs, and atIBDVs. The results revealed four typical mutations (G24, I141, V163, and E240) in VP1, two typical mutations (K221 and I252) in VP2, two typical mutations (Q167 and L196) in VP3, and five typical mutations (R7, P44, R92, G104, and E147) in VP5, with no typical mutations in VP4. As currently used vaccines in China could not provide complete protection against the novel variant IBDV, it could be speculated that the change of antigenic region in VP2 might be an important reason (21). Antigenic drift is an important factor that affects the protective efficiency of vaccines against field strains (25). Previously, it has been shown that the variant strain isolated in the USA exhibited antigenic drift which further affects the neutralizing epitopes in VP2 (26). In this study, the analysis of the antigenic index of the HVR of VP2, the only protein known to induce neutralizing antibodies against IBDV, demonstrated that there were three regions that showed a different antigenic index in the novel variant strain compared with other genotypes of IBDV (Supplementary Figure S3). However, the functions of these mutations and the role of each different antigenic region were rarely studied, further research will focus on the study of them.

In conclusion, a strain of IBDV, designated HB202201 was isolated from young laying hens, and full-length genome sequences of segment A and segment B were analyzed. The phylogenetic analysis of the segment A and segment B of HB202201 showed that the newly isolated strain belonged to A2dB1 genotype. Furthermore, multiple amino acid specific mutations in IBDV-encoded proteins and different antigenic regions in the HVR of VP2 were found in the novel variant IBDVs. All these data will contribute to the understanding of the genomic characteristics of the novel variant strain and its vaccine development.

## Data availability statement

The original contributions presented in the study are publicly available. This data can be found at: https://www.ncbi.nlm.nih.gov/; OR290134-OR500109.

## Ethics statement

The animal studies were approved by the Institutional Animal Care and Use Committee of Yangzhou University. The studies were conducted in accordance with the local legislation and institutional requirements. Written informed consent was obtained from the owners for the participation of their animals in this study.

## Author contributions

ZB: Conceptualization, Writing—original draft. SW: Data curation, Investigation, Writing—original draft. KX: Data curation, Investigation, Writing—original draft. CZ: Supervision, Writing—review & editing. MG: Supervision, Writing—review & editing. YC: Supervision, Writing—review & editing. XZ: Funding acquisition, Writing—review & editing. YW: Funding acquisition, Writing—review & editing.

## Supplementary material

Supplementary Figure S1 Sequence alignment of amino acids of the HVR in different Chinese novel variant IBDVs. The HVR amino acid of 10 strains of novel variant IBDVs were aligned using DNAMAN software.

Supplementary Figure S2 Phylogenetic analysis of the full-length of segment A and segment B. Phylogenetic analysis of the full-length of segment A from 44 strains of IBDV **(A)**, and the full-length of segment B from 32 strains of IBDV **(B)**. Both phylogenetic trees were constructed using the maximum likelihood (ML) method by MEGA-X software.

Supplementary Figure S3 Antigenic index plots of the amino acid sequences of the HVR of VP2. 10 representative IBDV strains with different genotypes were chosen for the analysis of the antigenic index. The antigenic index plots were analyzed using the DNASTAR software using Jameson-Wolf algorithm. Above-zero levels indicate predictive antigenic sites, and changes in the antigenic index in the novel variant strain are labeled with rectangles.
