# Supplementary figures and images for: Isolation and genomic characteristics of the novel variant infectious bursal disease virus in China
Source: Front Vet Sci. 2023 Dec 11;10:1314903. doi: 10.3389/fvets.2023.1314903 (PMC10749300; doi:10.3389/fvets.2023.1314903)

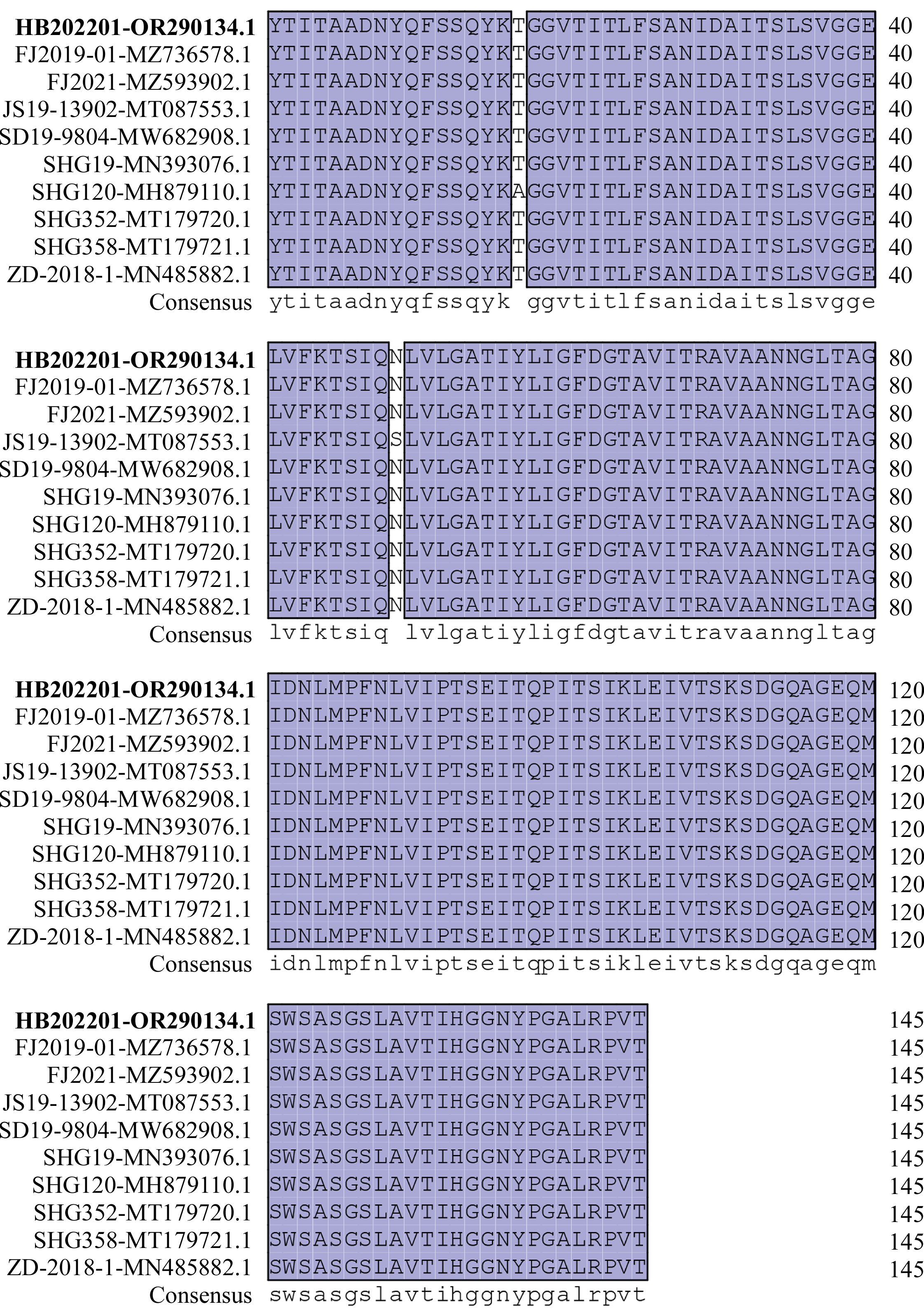

Supplement: Supplementary Figure S1 — Sequence alignment of amino acids of the HVR in different Chinese novel variant IBDVs. The HVR amino acid of 10 strains of novel variant IBDVs were aligned using DNAMAN software. [file Image_1.TIF]

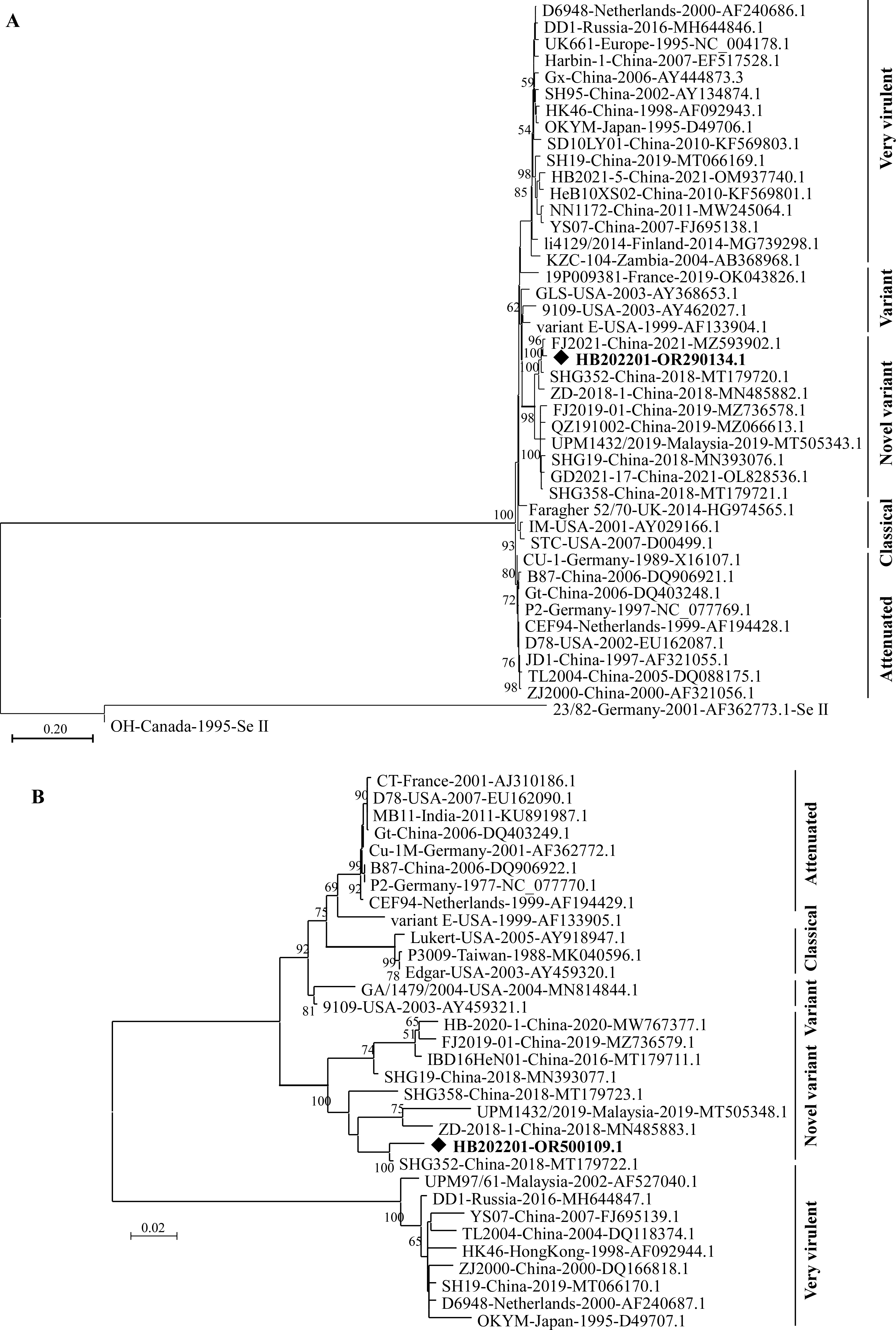

Supplement: Supplementary Figure S2 — Phylogenetic analysis of the full-length of segment A and segment B. Phylogenetic analysis of the full-length of segment A from 44 strains of IBDV (A), and the full-length of segment B from 32 strains of IBDV (B). Both phylogenetic trees were constructed using the maximum likelihood (ML) method by MEGA-X software. [file Image_2.TIF]

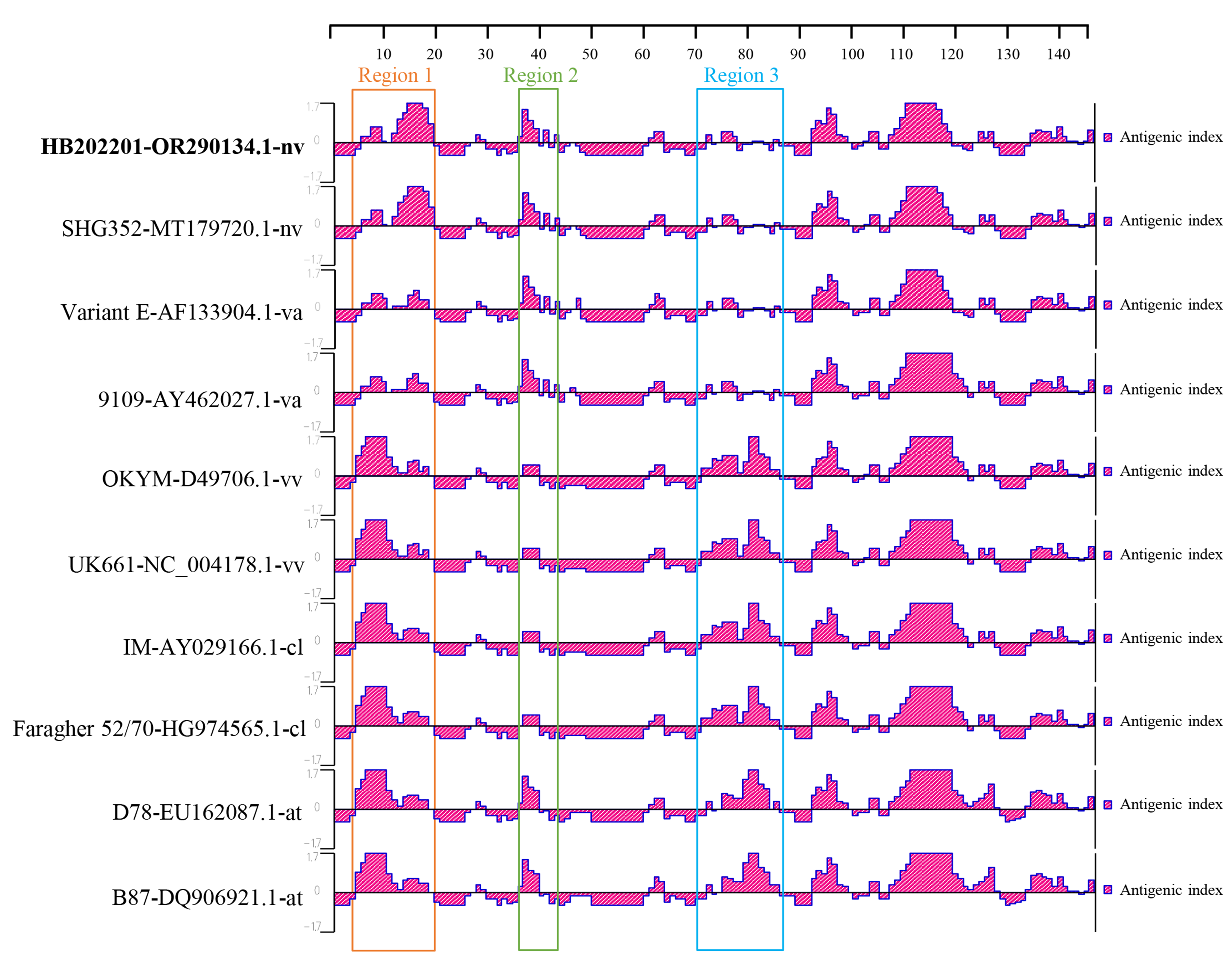

Supplement: Supplementary Figure S3 — Antigenic index plots of the amino acid sequences of the HVR of VP2. 10 representative IBDV strains with different genotypes were chosen for the analysis of the antigenic index. The antigenic index plots were analyzed using the DNASTAR software using Jameson-Wolf algorithm. Above-zero levels indicate predictive antigenic sites, and changes in the antigenic index in the novel variant strain are labeled with rectangles. [file Image_3.TIF]
